# Web-Based Prescription Opioid Abuse Prevention for Adolescents: Program Development and Formative Evaluation

**DOI:** 10.2196/12389

**Published:** 2019-07-19

**Authors:** Sarah K Moore, Michael Grabinski, Sarah Bessen, Jacob T Borodovsky, Lisa A Marsch

**Affiliations:** 1 Center for Technology and Behavioral Health Geisel School of Medicine Dartmouth College Lebanon, NH United States; 2 Geisel School of Medicine Dartmouth College Hanover, NH United States; 3 Health and Behavior Research Center Department of Psychiatry Washington University School of Medicine St Louis, MO United States

**Keywords:** opioids, prevention and control, adolescent, randomized controlled trial, internet

## Abstract

**Background:**

The unprecedented number of youths engaged in nonmedical use of prescription opioids (POs), as well as the myriad negative consequences of such misuse, emphasizes the importance of prevention efforts targeting this public health crisis. Although there are several science-based, interactive drug abuse prevention programs focused on preventing the use of nonprescription drugs in youths, to our knowledge, there are no science-based interactive programs that focus on the prevention of PO abuse among adolescents.

**Objective:**

The aim of this study was to develop and conduct a formative evaluation of a science-based interactive Web-based program focused on the prevention of PO abuse among adolescents aged 12 to 17 years (Pop4Teens). This study was conducted to prepare for a randomized controlled trial designed to evaluate the effectiveness of Pop4Teens compared with an active control website, JustThinkTwice.com (Drug Enforcement Administration), in impacting knowledge and attitudes about POs and perceptions of risk associated with the abuse of POs, as well as intentions to use and actual use of POs.

**Methods:**

We conducted 6 focus groups with 30 youths (a mean of 5 per group: the eligibility being aged 12-19 years) along a continuum of exposure to POs (in treatment for opioid use disorder, in general treatment for other substance use disorder, prescribed an opioid, and opioid-naïve) and writing sessions with 30 youths in treatment for opioid use disorder (12-19 years) to inform the development of the Web-based prevention tool. Feasibility and acceptability of a prototype of the Web-based intervention were then assessed through individual feedback sessions with 57 youths (drawn from the same populations as the focus groups).

**Results:**

We successfully completed the development of a Web-based PO abuse prevention program (Pop4Teens). Analyses of focus group transcripts informed the development of the program (eg, quiz content/format, script writing, and story editing). Selected writing session narratives anchored the planned scientific content by lending credibility and informing the development of compelling storylines intended to motivate the youth to engage with the program. Feedback session data indicated that the Web-based tool could be potentially useful and acceptable. In addition, feedback session participants demonstrated significant increases in their knowledge of key topics related to the prevention of PO abuse after the exposure to sections of the Web-based program.

**Conclusions:**

The opioid crisis is predicted to get worse before it gets better. An effective response will likely require a multipronged strategy inclusive of effective evidence-based prevention programs acceptable to, and accessible by, a majority of youths.

## Introduction

### Background

Nonmedical use of prescription opioids (NMUPO) among adolescents—use of a prescription without or inconsistent with a doctor’s order—is an escalating public health concern. Despite a plateauing of this type of drug use among youths in the past several years, 14.0% (2068/14,765) of a sample of high school students in a nationally representative study in 2017 endorsed lifetime misuse of prescription opioids (POs) [1]. Recent investigations underscore the gravity of the negative and potentially lifelong consequences associated with early-onset PO misuse. Strong associations between younger age of opioid initiation and heightened risk of opioid use disorders later in life have been demonstrated across a gamut of retrospective and longitudinal studies [2-4]. One study’s epidemiologic calculation suggests that for roughly 8 million adolescents (12-21 years) who engaged in NMUPO between 2002 and 2013, approximately 42,000 to 58,000 met the criteria for opioid use disorder— *during each year* —within 12 months after the onset of their nonmedical use [5]. This conversion rate from experimentation to opioid use disorder reveals the addictive potential of this class of drugs. In addition, a prospective nationally representative study of high school seniors demonstrated that prescribed opioid use before high school graduation is independently associated with a 33% increase in the risk of future opioid misuse after high school. Notably, this association is concentrated among youths who had little to no history of drug use and disapproving attitudes toward illegal drug use at baseline [6].

NMUPO is also a strong predictor of heroin use onset among adolescents and young adults [4,7-9]. In a national study of youth aged 12 to 21 years, those who reported past NMUPO had a 13-fold increased risk of heroin initiation compared with those with no previous history of such use [10]. Young people who use POs nonmedically are also significantly more likely to experience major depressive episodes (MDEs) [11], as well as sexual violence [9,12], compared with their nonabusing peers.

Further underscoring the urgent need for prevention of NMUPO among the youth, recent statistics show that drug overdose is now the leading cause of death for adults aged under 45 years in the United States, driven largely by opioid-related overdoses, which accounted for most of the 63,632 lethal overdoses in 2016 (65.99% (41,997/63,632) [13,14]. Between 1999 and 2016, the drug overdose death rate increased by 268.2% among those aged 15 to 19 years (N=7,921 adolescent deaths 1999-2016) [15]. Though the effects of introducing opioids to the developing brains of adolescents may be harmful in ways that are not yet well understood, the most immediate consequences are likely to impact cognitive, social, and emotional development [16,17]. For example, some data show that treatment-seeking adolescents with opioid use disorder demonstrate working memory impairment [18], as well as daily drug using/seeking routines that leave little room for healthy social development and alternative interests [19]. In addition, in a recent study using a nationally representative dataset, youths who engaged in NMUPO were 120% more likely to experience an MDE in their lifetime [20]. The current National Institutes of Health (NIH) initiative, the Adolescent Brain Cognitive Development Study [21], designed to prospectively study the risk and protective factors influencing substance use and its consequences among a cohort of 9- to 10-year-olds (for 10 years) will likely add much to our understanding of developmental consequences. The number of youths currently engaged in NMUPO, as well as the possible consequences of such misuse, unequivocally emphasizes the importance of prevention strategies targeting this public health crisis.

Nationwide efforts to provide a comprehensive response to this crisis include the following: the development and deployment of interventions such as prescription drug monitoring programs to reduce inappropriate prescribing of opioids and enable the early identification of persons demonstrating problematic use [22], increasing the number of prescribers who receive training on pain management and safe opioid prescribing [23-25], and expanding access to abuse-deterrent formulations to discourage abuse [26]. There are also numerous efforts underway to improve access to, and insurance coverage for, evidence-based treatment for individuals with opioid use disorders [25,27]. The availability of the opioid antagonist naloxone hydrochloride (NARCAN), which reverses the potentially fatal respiratory depression caused by heroin and other opioids, is increasing among law enforcement and laypersons alike across the country [28]. However, limited attention has been given to the development of evidence-based prevention programs to deter opioid misuse among the youth. The prevention program that we have developed and plan to evaluate in an upcoming randomized controlled trial is the first of its kind, to our knowledge, as existing prevention programs do not incorporate scientific knowledge about risk factors for PO abuse.

Risk factors for PO abuse differ from other drugs/illegal drugs in significant ways: the vast majority of nonmedical PO users report obtaining these medications for the first time from friends or family [29], lowering the threshold to access in terms of both response effort and costs; the perceived risks (eg, health and legal) associated with the misuse of these medications are significantly attenuated compared with other drugs of abuse because of their prescription and approval for medical use [30,31]; and opioid prescriptions for acute pain episodes set the occasion for either the misuse of leftover medications [6,32,33] or enticement of those youths who are prescribed opioids into diverting (eg, selling, trading, and sharing) their medication for monetary or social gain [34].

Irrespective of the specific risk factors associated with the use of a particular class of drugs, effective drug abuse prevention programs generally educate the youth about how specific drugs work (mechanism of action) and typically focus on one of 2 broad categories of skill training [35]: social influences and training skills necessary to resist peer, family, media, and other social influences known to promote drug use [36] and the provision of more general skills training necessary for social competency and the ability to cope with stressful life situations [37]. Numerous qualitative and quantitative reviews of the prevention science literature have demonstrated that substance abuse prevention programs based on these approaches generally increase protective factors, reduce risk factors, and produce marked reductions in drug use among the youth [38]. In addition, programs based on social influence and general skills training models have been shown to be effective when delivered via interactive, activity-oriented programs and not traditional, didactic instructional techniques [39,40].

Digital platforms may provide a particularly appealing and effective approach for prevention efforts designed to reach adolescents. The traditional means of reaching the youth are hindered by the costs associated with teacher/clinician-delivered interventions as well as manifold issues that impede delivering interventions with fidelity. However, interventions delivered via digital platforms (eg, laptop, mobile phone, or computer) boast of benefits that have greater appeal to the youth [41], significantly reduced costs [42], standardized content delivery [42], the potential for delivery across a multitude of settings [43], and minimizing teaching and/or clinical staff burden compared with traditional methods [44]. Underscoring the opportunity afforded by digital platforms, as of 2018, 93.9% (698/743) of teens reported going on the Web daily—including 44.9% (334/743) who said they go on the Web *almost constantly*. These numbers are largely a function of mobile device usage and/or ownership with nearly 95.0% (706/743) of teens endorsing one or the other [45].

Published scientific literature on digital platforms for interventions targeting adolescent substance abuse is burgeoning [41,46].The number of publications describing technology-based substance abuse prevention programs that aim for universal application, that is, intended for all youths who endorse no past alcohol or substance use, is significantly smaller. These programs have demonstrated effectiveness in 3 contexts: primary care [47], schools [48,49], and homes [50-53]. They target the prevention of different classes of drug use, for example, alcohol [49,50,54], cannabis [49,51], and tobacco [48] and generally comprise interactive internet-based activities that function to increase drug-related knowledge and shape user attitudes and normative beliefs around substance use in ways that result in abstinence or delayed onset of use [51]. All programs referenced herein were found to be effective in reducing intentions/expectations to use and/or endorse use.

Thus, although several science-based interactive drug abuse prevention programs have been developed to prevent the use of nonprescription drugs in youths, to our knowledge, there are no published studies featuring science-based interactive programs focused on the prevention of PO abuse among adolescents. As described below, the scientific evidence underpinning the Web-based adolescent prevention program content that we described in this paper comes from the literature on computer-delivered interventions [41,55,56], computer-assisted instruction technology [57-59], and identified risk factors for PO use among the youth [5,6,8,9,30,33,60].

The data presented in this paper are from a completed Phase I of a Small Business Technology Transfer (STTR) grant from the National Institute on Drug Abuse (NIDA; R41DA023731) as well as a NIDA STTR Phase II grant (2R42DA023731–02). These efforts extend our previous study, developing and evaluating science-based digitally delivered substance abuse prevention programs for the youth, with a focused goal to prevent PO misuse among the youth.

### Aims

The aim of the research reported herein was to develop and conduct a formative evaluation of a science-based Web-based interactive program focused on the prevention of PO abuse among adolescents. This program is composed of 8 modules addressing the following topics: What are POs?; misconceptions that POs are safe and nonaddictive; misconceptions that using POs without a prescription is not illegal; risks of PO misuse; nonmedication alternatives for pain management; refusing offers to misuse POs; refusing requests by others for a PO prescribed to you; and how to know if you or someone you know may be addicted. This study was conducted before conducting a randomized controlled trial to evaluate the effectiveness of the Web-based PO abuse prevention program, Pop4Teens, compared with an active control, the JustThinkTwice.com website (Drug Enforcement Administration), in impacting attitudes about, knowledge and perceptions of risk associated with the abuse of POs, as well as intentions to use and actual use of POs. The results of the randomized controlled trial will be published separately once the trial is completed.

## Methods

### Design

Our iterative development process spanned Phase I (when 3 modules were developed, ie, *introduction*, *what are POs*, and *misconceptions that POs are safe and nonaddictive*) and Phase II (when the remaining modules were developed—see above). We did not include the *introduction* module in the final product as it had become redundant because of the inclusion of the content of subsequent modules. These activities included the conduct of the following: 6 focus groups with youths along a continuum of exposure to POs (in treatment for opioid use disorder, in general treatment for other substance use disorder, prescribed an opioid, and opioid naïve); writing sessions with youths in treatment for opioid use disorder to inform the development of the Web-based prevention tool as well as to increase the overall credibility of the content; and one-on-one feedback sessions with youths (drawn from the same populations as the focus groups) *.* We conducted focus groups to help shape program content development and feedback sessions to obtain systematic feedback on the beta version of the Web-based intervention.

### Participants

Participants for focus groups and individual feedback sessions were recruited from a treatment program focused on the treatment of opioid-dependent youths, a community-based adolescent substance abuse treatment program, and the community via advertisements/flyers for Phase I and either a community hospital setting or a treatment facility for opioid-dependent adolescents and young adults for Phase II. Participants for the Phase II writing sessions were recruited exclusively from the latter. Participants in focus groups and writing and feedback sessions in both Phases I and II were compensated $30 for their time (see Table 1 for details on activities, eligibility criteria, and sample sizes).

**Table 1:** Overview of aims and participant activities by phase.

Small Business Technology Transfer^a^ Phase – Aim	Primary aims	End user activity	Eligibility criteria	Sample size (n)
I – 1. (2010-2011)	Develop pilot content of a Web-based PO^b^ abuse prevention program for High school-aged Youth	FG^c^ 1	Youth 14-18: not in substance abuse treatment / opioid naive	6
		FG 2	Youth 14-18: in treatment for wider substance abuse issue	5
		FG 3 (Interview)^d^	Youth 14-18: in treatment for opioid dependence	1
		Individual feedback sessions	Youth 14-18: along a continuum of exposure to opioids (paralleling groups from FG 1, 2 and Interview)	30
II – 1. (2014-2016)	Complete development of ALL components of a Web-based PO abuse prevention program/Integrate all components into a unified, Internet-based multimedia package to be run cross-platform	FG 1	Youth 12-19^e^: in treatment for prescription opioid dependence	8
		FG 2	Youth 12-17^f^: not in substance abuse treatment/ opioid naïve	6
		FG 3	Youth 12-17: prescribed an opioid in the past year	4
		Writing sessions	Youth in treatment for opioid dependence	30
		Individual feedback sessions	Youth 12-19: along a continuum of exposure to opioids (paralleling groups from FG 4, 5 and 6)	27
II – 2. (2017-2018)	Conduct a randomized, controlled trial to evaluate the effectiveness of a Web-based PO abuse prevention program	Randomized, controlled trial	Youth 12-17, English literate, whose parents provide consent (w/ access to Internet)	400 (planned)

^a^A program that expands funding opportunities in the federal innovation research and development arena.

^b^PO: prescription opioid.

^c^FG: focus group.

^d^We were able to recruit only 1 adolescent who met the eligibility criteria and consequently conducted an in-depth interview instead of a focus group.

^e^The eligible age range was broadened for youth in treatment for PO dependence due to the fact that most youth do not enter treatment for opioid dependence before 16 years of age.

^f^The target age range for Phase I (14-18) was determined to be too old in light of scientific literature related to prescription opioid misuse among youth published between the funding of Phases I (2010) and II (2014). In the interests of (1) locating our work within the larger corpus of effective prevention efforts targeting adolescent substance abuse in general (Hale et al, 2014), (2) attending to epidemiological work (Meier et al, 2012) cautioning against designing prevention initiatives that focus on the later high school years, and (3) seeking to help youth on the younger tail of initiation of prescription opioid abuse avoid the potentially significant negative long-term consequences of early experimentation (McCabe, 2007), we adjusted the range to youth between the ages of 12-17 for Phase II.

### Procedure

All relevant institutional review board approvals were obtained before the commencement of each phase of the research. Adolescents' parent/guardian provided consent for their child (if aged <18 years) and adolescents provided assent (if aged <18 years) or consent (if aged ≥18 years) to participate (with the exception of the writing session, for which we obtained a waiver of assent/consent because of its anonymity).

#### Focus Groups

We conducted the first series of 3 audiotaped 90-min focus groups with the youths to determine how to best present the information in the program to the target age group. The youths provided input on all aspects of the program content, including the structure and style of the program. Participants were asked to brainstorm and systematically fine-tune the various components of this program during these focus groups, which were conducted before the planned pilot program content was developed.

Focus groups were conducted in a semistructured group interview format, in which the content of the discussion was guided by a list of key topics relevant to the 3 modules developed in this phase of the development of the tool (eg, *What are POs?*; *What do you think they do?*; and *Why do you think doctors prescribe opioid medications?*). The youths were asked a series of questions regarding the extent to which the language, video, characters, graphics, and presentation style and structure to be used in presenting the desired material (described below) were (1) understandable, (2) engaging, and (3) relevant to their experience and the experience of those their age. All data obtained from focus groups were qualitative in nature and were used to shape program development in a manner that is developmentally appropriate for and acceptable to the target audience.

The second series of 3 focus group discussions (designed to inform the development of the remaining 6 modules using a similar process as described in the previous section) were structured by questions probing the following: misconceptions that using POs without a prescription is not illegal, risks of PO misuse, and how to know if *you or someone you know* is addicted (focus group 1: youths in treatment for opioid dependence); assessments of credibility and compelling nature of stories written by youths in treatment (focus group 2: opioid-naïve youths); and what teens need to know if prescribed an opioid (physical, emotional, and social effects), impact of experience on the future use of POs, and how to respond if approached to share POs (focus group 3: youths prescribed an opioid).

#### Writing Sessions

To strengthen the credibility of the prevention program, we recruited youths in treatment for their opioid dependence from a therapeutic substance abuse treatment center to write stories describing topics of potential interest to program users, as well as topics paralleling planned scientific content: their lives just before using POs the first time, their motivations to use POs the first time, how quickly the time elapsed between using POs the first time and then losing control of their use, their experiences of realizing that they had a problem with POs, and also, how to know if you or someone you know is addicted to POs. The need to protect the writers’ anonymity constrained the content of the narratives. Treatment center staff led the writing sessions to ensure no stories revealed the identities of the authors or those of others around them. Writing sessions occurred in small groups at times that were convenient for them throughout a day of their treatment experience. They were instructed to choose a pseudonym to put at the top of their paper, along with age and race, and to tell their story *without risking the identity of friends or family members*. There were no time limits on the writing sessions other than those naturally imposed by treatment program schedules. The staff reviewed the narratives for any identifying information before sharing the anonymous files with the research team. Our team used some of these stories to anchor the Web-based program, as well as to generate scripts for actors to portray some of this content in video clips conveying planned content (eg, risks of PO use and refusing requests from others for a PO prescribed to you). Input from the focus groups, as well as the stories that best fit with the planned content (eg, risks associated with opioid misuse), was incorporated into the development of the beta version of the Web-based program.

#### Feedback Sessions

During the iterative development process in each phase, we also conducted one-on-one feedback sessions with the youths to (1) enable them to access the program modules on a study phone, tablet, or laptop and (2) systematically provide feedback on the beta version of the Web-based program. After completing a brief demographic form, participants completed a 2- to 5-item presession knowledge test to probe baseline familiarity with the content of the section topic to be reviewed. An example of a multiple choice question from a knowledge test is, “Prescription opioids vary in strength and the effects are ‘dose related,’ meaning ______________________.”

Participants then completed the Web-based module at their own pace, answered the knowledge test items again, and completed a brief 17-item feedback survey inclusive of 12 visual analog scale (VAS) items and 5 open-ended response items. Possible values for VAS scores ranged from 0 to 100 mm and were anchored in terms of the variable of interest probed in the item. For example, for the first item—“How interesting was the section of the program you just completed?”—0 was anchored by the phrase *not interesting*, whereas 100 was anchored by *very interesting*. Most participants reviewed 2 modules in a single feedback session and completed separate pre- and postsession knowledge tests and feedback surveys for each module (see Multimedia Appendix 1 for the 17-item feedback survey).

### Analysis

We transcribed the focus group audiotapes and reviewed the transcripts while listening to the audiotapes to ensure accuracy. The focus group data were thematically analyzed to inform the development of the program. For the feedback sessions, mean VAS scores were calculated for each item. Paired *t* tests were used to compare pre- and postsession knowledge test accuracy data, collapsed across modules and participants. Feedback session data were used to inform program refinement before the launch of the trial, for example, feedback on program content and interactive features.

## Results

### Participant Characteristics

Participants across development activities were predominantly non-Hispanic white males. Of those queried, only a small handful had previously participated in any drug prevention program geared toward POs. Of the youths in treatment for opioid dependence use disorder, the average age of first opioid use was 14.7 years (SD 1.2) for youths in focus groups and 15.75 years (SD 2.4) for youths in writing sessions and half had transitioned to heroin use before seeking help with their opioid use (see Table 2 for more detail).

**Table 2:** Program development participant’ demographic characteristics.

Characteristic		Phase I		Phase II		
		Focus groups #1-3 (n=12)	Feedback sessions (n=30)	Focus groups #1-3 (n=18)	Feedback sessions (n=27)	Writing sessions (n=30)
Age (years), mean (SD)		16	16.53 (1.1)	16.77 (2.2)	16.37 (2.2)	21.83 (2.0)
**Gender, n (%)**						
	Female	5 (42)	13 (43)	11 (61)	11 (41)	9 (30)
	Male	7 (58)	17 (57)	7 (39)	16 (59)	21 (70)
**Ethnicity, n (%)**						
	Non-Hispanic	—^a^	27 (90)	16 (89)	26 (96)	13 (87)^b^
**Race, n (%)**						
	White	—	15 (50)	15 (83)	24 (88)	—
	Black	—	6 (20)	—	1 (4)	—
	Mixed/other	—	9 (30)	3 (17)	2 (8)	—
Previous experience w/ PO^c^ Drug Prevention Program, n (%)		1 (7)	1(3)	—	1 (4)	—
Age at first PO use, mean (SD)		—	—	14.7 (1.2)^d^	—	15.75 (2.4)
Transition from PO heroin use, n (%)		—	—	6 (86)^d^	—	12 (41)

^a^Data not collected.

^b^Denominator is 15.

^c^PO: prescription opioid.

^d^Applies only to youth in focus group #1 (n=8).

#### Focus Groups

The first series of 3 focus groups were focused on gleaning an understanding of what the youths know about POs, as well as misconceptions that POs are safe and nonaddictive. The youths were generally very well informed about the indication for POs. However, they were equally uninformed in their assessment of their addictive potential and associated risks of overdose. Analysis of data from the second set of focus groups among the youths in treatment for opioid dependence highlighted the following themes: the latency between first use of opioids and loss of control is so brief that most youths report not even noticing (range: 3-8 months); self-medication of unwanted feelings, combined with low threshold access to prescribed medication, is the primary reason for many youths to first use POs; and POs are perceived as the second most dangerous drug (next to heroin) among the youth who have experimented with many substances. The transcript data from the second focus group of opioid naïve youths suggest that based on their reading of the stories written by the youths in treatment, they believe the program should avoid moralizing; *laundry-list-style* detailing of negative consequences; and normalizing other drug use when highlighting the severity of prescription and other opioid (eg, heroin) abuse. The analysis also suggests that the youths believe the program should include stories of how *average* kids have gotten into trouble with POs to highlight the nondiscriminatory dimension of opioid abuse among the youth (eg, a star athlete and a kid from a happy family).

Finally, the youths prescribed an opioid in the past year who participated in a focus group reviewed the refusal skill scripts drafted by research team members and provided written edits. The youths provided valuable insights on how to increase the credibility of the refusal skill script for those who may want to know how to refuse requests by others for an opioid prescribed to *you*. Specifically, the youths feel that if anyone approaches you to share or sell your prescription pain medication, the overwhelming message needs to be one of consternation and affront that someone would consider asking for something that you need to manage your injury.

#### Writing Sessions

The writing session stories varied in length from 2 paragraphs to 5 pages of written text, single-spaced. Members of our research team identified narratives for program inclusion by comparing rankings based on the quality of the writing, as well as the extent to which narrative content matched planned content (eg, if a youth wrote about the challenges of saying *no* when offered a Percocet, this content parallels the planned module on how to refuse offers to misuse an opioid). Once the narratives were selected, we worked with a multidisciplinary media company to audition and identify actors to read the selected youth narratives to be recorded for program inclusion.

#### Feedback Sessions

**Figure 1:**
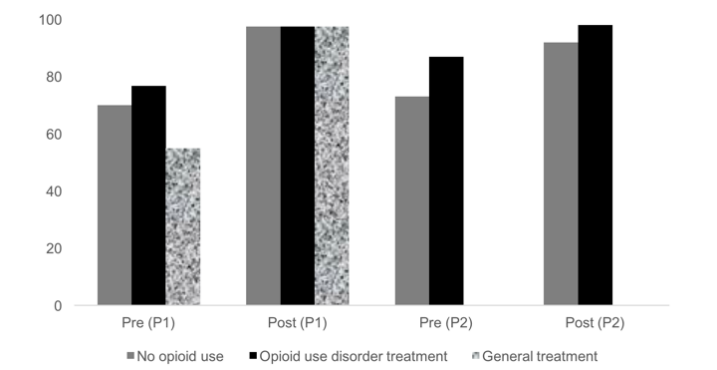
Pre- and postknowledge test scores: Phases 1 and 2 (P1, P2).

**Figure 2:**
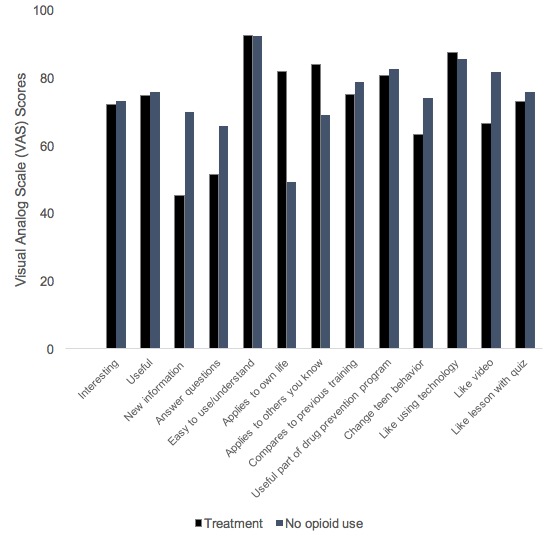
Feedback survey results.

Each of the participants recruited for individual feedback sessions reviewed 2 modules/program sections on one of the following platforms: mobile phone, tablet, or laptop. Feedback sessions were conducted individually, such that the participants individually interacted with the computer program. Scores indicate that participants made significant knowledge gains. For the modules developed in Phase I, the mean number of correctly answered items on the knowledge pre- and posttests was 3.65 (SD 1.46) and 4.4 (SD 0.75; *t*_19_=3.866; *P*<.001) for youths in treatment for opioid dependence, 2.5 (SD 1.27) and 4.4 (SD 0.68; *t*_19_=7.292; *P*<.001) for youths in general substance abuse treatment, and 3.1 (SD 0.72) and 4.4 (SD 0.75; *t*_19_=5.94; *P*<.001) for opioid naïve youths, respectively. For the 6 modules developed in Phase II, the mean number of correctly answered items on the knowledge pre- and posttests was 3.54 (SD 0.88) and 4.0 (SD 0.78; *t*_23_=−2.696; *P*<.05) for youths in treatment and 3.3 (SD 1.21) and 4.13 (SD 0.86; *t*_29_=−5.473; *P*<.001), for opioid naïve youths, respectively. See Figure 1 for a review of these data in terms of percent correct in the pre- and postknowledge test. Participants also rated the program sections positively. As shown in Figure 2, several of the responses to the 12 VAS items on the feedback session survey instrument fell between 80 and 100, indicating that participants found the modules to be *easy to use*, *understandable*, and *useful as part of a drug prevention program* and they also *liked using technology* and *liked the videos*. The remaining scale scores (eg, *interesting*, *useful*, *new information*, *answer questions*, *applicable to others you know*, *comparable to other information or treatment on this topic*, and *helpful [in terms of] change[ing] behavior*) fell between 60 and 80, with the exception of 1 item that probed the applicability of the content to one’s own life. This item had a mean score of 49 among opioid naïve youths (SD 27.03), which is not surprising given that the program is anchored by stories of youths in treatment for opioid dependence. In addition to the VAS items, participants were asked the following open-ended questions: “What did you like most about the program?”; “What did you like least?”; and “What suggestions might you make to improve the program?”. Participants in both groups overwhelmingly liked the *stories* (eg, “I mainly liked the person story about a young adult that actually experienced an addiction problem. It makes the program more real because it is based on a real person”) and *videos* (eg, “I liked the three videos I viewed because at times when they were a bit corny and funny, they got the point across in a relatable and effective way”) the best. The youths in treatment ranked the stories as most preferred, and opioid naïve youths ranked the videos as most preferred but each group predominantly referenced these 2 features as the *best*. What the youths liked least was unanimous: the *quizzes* were least liked across all groups of youths (eg, “I liked the quiz the least because it kept asking the same questions”). Suggestions for improvement were largely feedback on how to improve the quizzes (eg, “Don’t’ time the questions” and “Make quizzes less repetitive”).

### Development Product: Pop4Teens Program

On the basis of the information gleaned from the development activities, our technology team completed the final version of the Web-based intervention to be run cross-platform (ie, on mobile devices, tablets, and laptops). The 4 cornerstones of the Pop4Teens program are as follows: 8 stories of real youths in treatment for opioid use disorders and their companion lessons, videos, and quizzes. The stories provide an entrée to the program and potentially strengthen the credibility and compelling nature of the content by offering real-world experiential information on motivations to use POs for the first time, how quickly the time elapses between using POs the first time and losing control of use, experiences of realizing that the youths had a problem with POs, and how to know if you or someone you know are addicted to POs (see Multimedia Appendix 2). Lessons are bullet-point scientific summaries that accompany individual stories to reinforce content (see Multimedia Appendix 3). A total of 6 short (approximately 5-min) videos were the products of scripts that we created based on planned skill-building content as well as feedback from focus group youths. Working with a multimedia production company, we auditioned and identified actors to act out 3 separate skill-based scenarios: how to refuse offers to misuse opioids, how to refuse requests by others for a PO prescribed to you, and how to nonmedically manage pain through controlled breathing and relaxation exercise. In addition, the program includes 2 informational videos (*What are prescription opioids?* and *Misconceptions that prescription opioids are safe and nonaddictive*) developed during Phase 1 of the STTR grant mechanism (see Multimedia Appendix 4). Finally, the lessons provide the source material for the quiz questions (see Multimedia Appendix 5).

## Discussion

### Principal Findings

Though time consuming, there is no substitute for enlisting potential end users in the development activities for Web-delivered intervention programs, for example, user-centered design [61] and behavioral science–informed user experience design [62,63]. A key element in the development of new technologies for behavior change is whether it suits its purpose and meets users’ needs and expectations [64]. Through the engagement of youths along a continuum of exposure to opioids through focus groups and writing and feedback sessions, Pop4Teens reflects the perspectives of these youths with the goals of maximizing acceptability, engagement, usability, utility, relevance, credibility, and, ultimately, effectiveness in reaching program outcomes.

Focus group input helped shape the Pop4Teens program in significant ways, several of which warrant underscoring. First, the research team had chosen select stories produced by the youths (ie, youths in treatment on their loss of control) for inclusion in the program. However, after enlisting the input of opioid naïve youths on these top-ranked stories, several were swapped for alternatives that were less *laundry-listy* of negative consequences or edited to remove what opioid-using youths found to be the normalization or minimization of other drug use. Second, the importance of the input from youths who had been legitimately prescribed an opioid that directed the emotional tone of how to respond if/when a friend requests to use medication prescribed to them for an injury cannot be overstated. The participants in this focus group were unanimous in their feeling that the response needs to be one of indignation and affront because in their collective experience, most friends/acquaintances do not accept *no* for an answer and often persist in their pressure to divert medications. This information was critical in the development of the skill-based video on the same topic. Third, the pervasive input from youths in treatment for opioid use disorder on the latency between first use and loss of control of PO misuse functioned to make this a point that we highlight repeatedly in the program. The youths in this focus group wanted to make sure we conveyed strongly that these drugs are not similar to other drugs of experimentation because of their addictive potential and loss of control.

The decision to enlist youths in treatment to share their stories was based on the voluminous literature on the social learning theory [65] which suggests that behavior change resulting from interactions with peers may be more likely than interactions with others (eg, in this case, adults, experts, and scientists) because peers are perceived to be more credible role models and enhance self-efficacy. Including youths in treatment to write about their experiences of losing control of PO misuse as part of the development process ultimately served a dual purpose. On the one hand, feedback session data support the stories’ inclusion to increase engagement with the program. On the other hand, the youths who provided their stories widely reported being grateful for the opportunity to turn a very dark period in their lives into something potentially positive and lifesaving for other youths. Finally, feedback session data demonstrate the promise and potential effectiveness of a Web-based approach to preventing opioid misuse among teens, given the statistically significant increases in participant knowledge between pre- and postsession module reviews as well as the overall positive assessment of the program.

The widespread use of technology among the youth highlights the important opportunities for delivering effective technology-based universal prevention interventions such as Pop4Teens to this group. Applicability of this technology-based tool may include its use across a range of settings, including primary care [48], schools [50,66,67], and individual homes [52,54,68-70]. In the primary care setting, Pop4Teens could conceivably be suggested to the youth as part of universally targeted care approaches within primary care practices across the country. For example, once a young person turns 12 years of age, the program could be part of a package of behavioral health recommendations that are considered part of the annual visit and thus recommended until the youth turns 17 years of age. In school settings, where most prevention interventions are delivered, Pop4Teens could be adopted as a stand-alone unit on POs (especially in communities hardest hit by the epidemic) or could be used in conjunction with other intervention content to expand the current drug prevention programming. Finally, the Pop4Teens tool might conceivably become a tool that parents and youths could access for free without barriers, off the internet.

### Conclusions

The opioid crisis is predicted to get worse before it gets better [71]. An effective response will likely require a multipronged strategy inclusive of improved regulation and monitoring of opioid prescribing, increased support for effective nonopioid approaches to pain management, and increased availability of affordable, evidence-based treatment options for the millions of Americans battling opioid use disorders. Effective, evidence-based prevention programs to help young people steer clear of the enormous sink hole into which tens of thousands have stumbled, including those who will not emerge, and the others who struggle mightily to claw their way out are also needed. Pop4Teens is the first science-based interactive Web-based program focused on the prevention of PO abuse among adolescents. Results from an ongoing randomized controlled trial evaluating Pop4Teens will help determine whether a tool of this type is effective in the prevention of PO misuse among adolescents. We plan to publish outcome data upon completion of the trial in 2019.

## Appendix

Multimedia Appendix 1 Feedback session survey.

Multimedia Appendix 2 Stories.

Multimedia Appendix 3 Lessons.

Multimedia Appendix 4 Videos.

Multimedia Appendix 5 Quizzes.

## Abbreviations

MDE: major depressive episode
NIDA: National Institute on Drug Abuse
NIH: National Institutes of Health
NMUPO: nonmedical use of prescription opioid
PO: prescription opioid
STTR: Small Business Technology Transfer
VAS: Visual Analog Scale
