# Supplementary material for: Web-Based Prescription Opioid Abuse Prevention for Adolescents: Program Development and Formative Evaluation
Source: JMIR Form Res. 2019 Jul 19;3(3):e12389. doi: 10.2196/12389 (PMC6676791; doi:10.2196/12389)
Supplement: Multimedia Appendix 1 [file formative_v3i3e12389_app1.doc]

**POP4TEENS**

**Feedback Form – Phase II**

**Story:_____________________________________________________**

**INITIALS STUDY ID DATE**

**INSTRUCTIONS:** Draw one line across the horizontal line at the point you think most ***honestly*** answers the question. When answering these questions, please think only about the section of the program ***that you just completed***.

1. How ***interesting*** was the section of the program that you just completed?

Make a mark on the line.

_______________________________________________________________________________________________

not interesting very interesting

2. How ***useful*** was the section of the program that you just completed?

Make a mark on the line.

_______________________________________________________________________________________________

not useful very useful

3. How much ***new information*** did you learn as a result of the section of the program that you just completed?

Make a mark on the line.

_______________________________________________________________________________________________

none a great deal

4. To what extent did the section of program that you just completed ***answer any questions*** you had about this topic?

Make a mark on the line.

_______________________________________________________________________________________________

not at all a great deal

5. How ***easy to use/understand*** was the section of the program that you just completed?

Make a mark on the line.

_______________________________________________________________________________________________

very difficult very easy

6. How much do you think this section of the program ***applies to*** ***your own life***?

Make a mark on the line.

_______________________________________________________________________________________________

not at all very much

7. How much do you think this section of the program ***applies to*** ***other people you know who are your age***?

Make a mark on the line.

_______________________________________________________________________________________________

not at all very much

8. How does this section of the program ***compare to any education or training sessions*** you have previously had on this topic?

Make a mark on the line.

_______________________________________________________________________________________________

This program is much worse. This program is much better.

9. How useful do you think this section of the program would be as ***part of a drug abuse prevention program for high school-aged teens***?

Make a mark on the line.

_______________________________________________________________________________________________

Not at all useful Very useful

10. Do you think this section of the program is ***likely to help high school-aged teens change their behavior?***

Make a mark on the line.

_______________________________________________________________________________________________

Not at all likely Very likely

11. How much did you ***like using the laptop, phone, tablet (circle the one you used)*** for this section of the program?

Make a mark on the line.

_______________________________________________________________________________________________

Not at all Very much

12. How much did you ***like the video*** in this section of the program?

Make a mark on the line.

_______________________________________________________________________________________________

Not at all Very much

13. How much did you ***like the section of the program where you were taught information and then quizzed on the information you learned?***

Make a mark on the line.

_______________________________________________________________________________________________

Not at all Very much

What did you like ***best*** about this section of the program?

What did you like ***least*** about this section of the program?

What ***suggestions*** do you have for changes to make to this section of the program or the program in general?

In what ***places*** do you think high school-aged youth might use this program?
