# Supplementary figures and images for: Web-Based Prescription Opioid Abuse Prevention for Adolescents: Program Development and Formative Evaluation
Source: JMIR Form Res. 2019 Jul 19;3(3):e12389. doi: 10.2196/12389 (PMC6676791; doi:10.2196/12389)

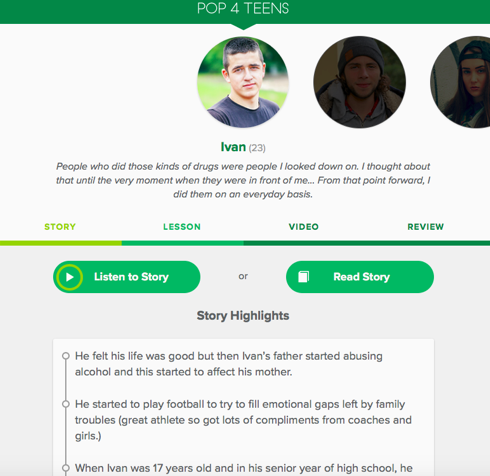

Supplement: Multimedia Appendix 2 [file formative_v3i3e12389_app2.png]

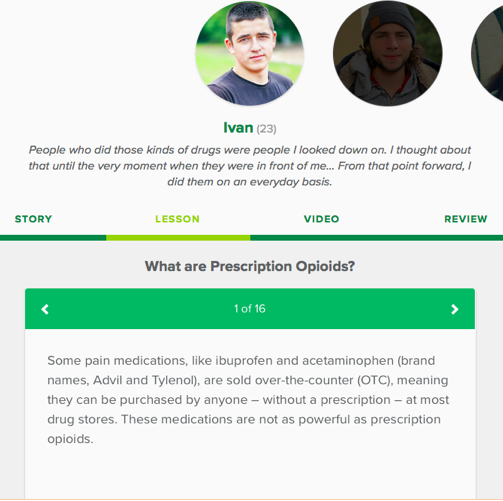

Supplement: Multimedia Appendix 3 [file formative_v3i3e12389_app3.png]

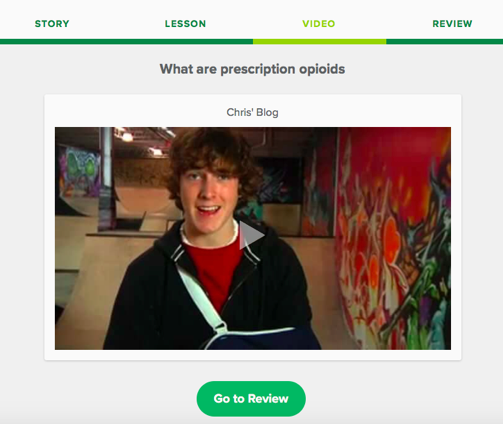

Supplement: Multimedia Appendix 4 [file formative_v3i3e12389_app4.png]

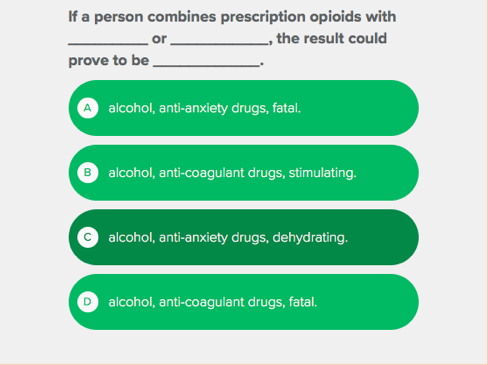

Supplement: Multimedia Appendix 5 [file formative_v3i3e12389_app5.png]
